# Effect of Oxidative Damage Due to Excessive Protein Ingestion on Pancreas Function in Mice

**DOI:** 10.3390/ijms11114591

**Published:** 2010-11-16

**Authors:** Chunmei Gu, Huiyong Xu

**Affiliations:** 1 Institute of Food Science and Engineering, Jilin Agricultural University, Jilin, Changchun 130118, China; 2 Jiutai City Traditional Chinese Medical Hospital, Jiutai, Jilin, Changchun 130500, China

**Keywords:** excessive protein diet, pancreas, oxidative damage, antioxidant, mice

## Abstract

The present study was undertaken to evaluate the effect of oxidative damage due to excessive protein diet on pancreas function in mice. For this purpose, thirty male (C57BL/6J) mice were randomly divided into three groups and fed on different diets as follows: group 1 was fed on a normal diet, group 2 was fed on an excessive protein diet and group 3 was fed on an excessive protein diet supplemented with 0.06 g/kg cysteamine. Each group was fed for 2 weeks, and then pancreas samples were collected to examine oxidative and antioxidant parameters and pancreas function. The results showed that ingestion of an excessive protein diet markedly increased contents of malondialdehyde (MDA) and decreased T-AOC and activities of antioxidants SOD and GSH-Px, compared with a normal diet (P < 0.05). Pancreas weight and concentration of protein, DNA and RNA were significantly higher (P < 0.05), digestive enzyme activities were significantly lower and levels of somatostatin and insulin were higher in mice fed with an excessive protein diet than those fed with a normal protein diet. In the group fed with excessive protein diet supplemented with cysteamine, oxidative stress was mitigated and pancreas function was improved. These data demonstrate that excessive protein ingestion could increase oxidative damage of free radicals on pancreas function through destroying the balance of oxidants and antioxidants.

## 1. Introduction

Nutrients may be one of the causative factors of oxidative stress. They cause redox imbalance and further lead to a number of diseases produced through accumulating reactive oxygen species (ROS) *in vivo* [1–3]. Protein is one of three major nutrients in organisms. Excessive protein ingestion can increase amino acid oxidation and urea synthesis [4] and decrease the nutritional efficiency of energy utilization [5]. However, the reoxidation of reducing equivalents derived from amino acid oxidation is linked to the mitochondrial redox chain [6]. Free radical generation during mitochondrial oxygen reduction may lead to oxidative stress if the antioxidant capacity is insufficient to quench the extra free radical production, and thereby endangers human health [7,8]. Gu *et al*. [9] found that a high-protein diet could destroy the balance of oxidation and antioxidants in the digestive system of mice and increase level of ROS in the digestive gland. The pancreas is an important glandular organ in the digestive system, and the normal operation of its function is essential for the digestion and absorption of nutrients. Moreover, many studies have confirmed that pathogeny of acute pancreatitis, which is one of the diseases with higher incidence, is attributed to the oxidative damage of free radicals on endocrine and exocrine function of pancreas [10,11]. In this regard, the present study was designed to investigate the oxidative damage of excessive protein ingestion on pancreas function in mice.

## 2. Materials and Methods

### 2.1. Animals and Diets

Male C57BL/6J mice (body weight, 12–13 g) were used in this study. All animals were housed under a controlled atmosphere (temperature, 23 °C ± 1; relative humidity, 55 ± 5%; and a fixed 12 h light:dark cycle, light 0700 to 1900 h). Prior to the feeding experiment, they were allowed free access to deionized water and a semipurified diet (Shanghai, China; crude protein 180 g/kg, crude fat 40 g/kg, metabolizable energy 11.9 MJ/kg) for 10 days to allow acclimatization to these conditions. Then all animals were divided randomly into three groups, each comprising of 10 mice: Group 1 (normal protein diet, NPD) received a normal diet containing 20% soy protein. Group 2 (excessive protein diet, EPD) received an excessive protein diet containing 60% soy protein. Group 3 received EPD supplemented with 0.06 g/kg cysteamine. Soy protein was exchanged isoenergetically by corn starch. The composition of the experimental diets is shown in Table 1. All mice were allowed free access to the experimental diets and deionized water throughout the experimental period. The care and use of the mice followed the institutional guideline of Jilin Agricultural University.

### 2.2. Sampling Procedures

At the end of the experimental period, mice were deprived of food overnight but had free access to deionized water. Mice were sacrificed by decapitation and the whole pancreas were removed immediately, gently rinsed in ice-cold PBS and then were cut into 50- to 100-mg portions as tissue samples. They were frozen in liquid nitrogen and stored at −80 °C for further treatment. After thawing, tissue samples were homogenized with ice-cold 0.9% NaCl solution and then were centrifuged at 4000 g for 15 min at 4 °C. The supernatants were used to determined content of protein, DNA and RNA, antioxidant defense and lipid peroxidation, digestive enzyme activities and hormone levels.

### 2.3. Analytical Methods

#### 2.3.1. Organ Index Determination

At the end of the experiment, body weight was determined and then mice were sacrificed by decapitation, the organs (including pancreas, liver, kidney and spleen) were removed and weighed, and organ indices were calculated according to the following formula:

Organ index=organ weight×100/body weight

#### 2.3.2. Lipid Peroxidation Determination

Lipid peroxidation products, thiobarbituric acid reactive substances (TBARS), were measured by a standard method and are expressed as the content of malondialdehyde (MDA) in nanomoles per milligram of protein [12].

#### 2.3.3. Antioxidant Activity Assay

Total superoxide dismutase (SOD) activity was assayed using hypoxanthine-xanthine oxidase-generated $O_{2}^{-}$· to reduce nitrotetrazolium (NBT) monitored spectrophotometrically at 550 nm.

Inhibition of NBT reduction to 50% of maximal is defined as 1 U of SOD activity and enzyme activity was expressed in units per milligram protein [13].

Glutathione peroxidase (GSH-Px) activity was measured according to the method of Hafeman *et al*. [14]. One unit of GSH-Px was defined as a decrease in the log of mmol GSH per minute and was expressed in unit per milligram protein. The automatic decrease of GSH without enzyme (control reaction under same condition) was subtracted from the calculation.

The T-AOC was measured by the method of Opara *et al*. [15].

#### 2.3.4. Protein, DNA and RNA Content Assays

Protein content was determined using the method of Lowry *et al.* [16].

DNA content was measured by the procedure of Giles and Myers [17]. In this procedure, glacial acetic acid containing 4% diphenylamine and 0.8 mg/mL acetaldehyde were added to DNA. Then DNA solution was incubated at 30 °C for 16 h, and then monitored spectrophotometrically at 595 nm.

RNA content in pancreas was assayed according to the method of Fleck and Begg [18] using an ultraviolet absorption assay.

#### 2.3.5. Digestive Enzyme Activity Assay

Amylase activity in pancreas was assayed using the method of Nelson [19].

Pancreatic lipase activity was measured according to the colorimetric method of Lowry and Tinsley [20]. In this method, a divalent metal copper was used and the copper complex was estimated spectrophotometrically at 440 nm.

Trypsin activity was measured by the method of Hummel [21] using TAME as substrate. Enzyme solution was mixed with 1 mM TAME in 10 mM Tris-HCl buffer, pH 8.0 and incubated at 30 °C for 20 min. Production of p-tosyl-arginine was measured by monitoring the increase in absorbance at 247 nm.

#### 2.3.6. Hormone Level Determination

Somatostatin and insulin levels were determined by the methods of Linda and Holst [22] and DeFronzo *et al.* [23], respectively, using commercial kits from North Immunoreagent Institute of Chinese Isotope Company.

### 2.4. Statistical Analysis

Data are reported as mean ± SD, *n* = 10. Differences between mean values were determined by ANOVA followed by comparisons using the Newman-Keuls multiple range test. Differences with *P* < 0.05 were considered significant.

## 3. Results

### 3.1. Viscera Indices in Mice

The EPD-fed group exhibited strikingly higher pancreas/body weight in mice compared to the NPD-fed group (Table 2), but other viscera indices had not significant change. Treatment with cysteamine strikingly lowered pancreas index (P < 0.05).

### 3.2. MDA Content, Activities of SOD and GSH-Px and T-AOC in Pancreas of Mice

Table 3 presents the effects of excessive protein diet on MDA content, activities of SOD and GSH-Px and T-AOC in pancreas. There was a significant increase in MDA content and significant decrease (P < 0.05) in SOD activity and T-AOC and decrease in GSH-Px activity of EPD-fed mice compared with NPD-fed mice. Supplement with cysteamine significantly decreased MDA content and significantly increased antioxidant capacity except GSH-Px activity of the EPD-fed mice.

### 3.3. Protein, DNA and RNA Content in the Pancreas of Mice

Feeding of the EPD led to significant changes of protein, DNA and RNA contents in pancreas, as is evident from Table 4. According to data, we found that protein, DNA and RNA contents were significantly increased by 21.69%, 20.37% and 72.14%, respectively, in the EPD-fed group compared to the NPD-fed group, which indicated that ingestion of excessive protein stimulated pancreas cell differentiation and growth. These levels were significantly lower in the group treated with cysteamine than in the EPD group (P < 0.05).

### 3.4. Amylase, Lipase and Trypsin Activities in Pancreas of Mice

Table 5 shows the effects of the excessive protein ingestion on digestive enzyme activity. There was a significant decrease (P < 0.05) in amylase, lipase and trypsin activities of EPD-fed mice compared with NPD-fed mice. Supplement with cysteamine increased digestive enzyme activity of EPD-fed mice and the activities of lipase and trypsin were significantly increased (P < 0.05).

### 3.5. Somatostatin and Insulin Levels in Pancreas of Mice

Feeding of the EPD for 14 days resulted in significant change of hormone level in the pancreas of experimental mice, as is evident from Table 6. There was a significant (P < 0.05) increase in somatostatin and insulin levels in pancreas of mice fed with EPD and EPD supplemented with cysteamine compared with mice fed with NPD. However, hormone levels were significantly lower in the EPD supplemented with cysteamine group than in the EPD group (P < 0.05).

## 4. Discussion

It is reported that a high-protein diet could result in an imbalance between oxidation and antioxidants in the digestive system of mice and increase the level of ROS in pancreas [9]. Oxidative stress is one of the causative factors of many diseases such as atherosclerosis [24]. An imbalance between production of free radicals and antioxidant levels leads to oxidative stress, which is obvious from the depressed antioxidant defense system in the EPD group of our study. Cysteamine acts as an antioxidant due to its sulfhydryl in relation to effectively scavenging free radicals (e.g., Hydroxyl radical) [25]. In the present study, administration of cysteamine to EPD-fed mice prevented the buildup of oxidative stress by restoring normal activities of the enzymatic antioxidants SOD and GSH-Px and normal level of the T-AOC in pancreas; the concentrations of these antioxidants were decreased in the EPD-fed mice. The diminished antioxidant defense system in EPD-fed mice leads to damage of the so-called lipid peroxidation system. We observed increased concentration of MDA, indices of lipid peroxidation, in pancreas of EPD-fed animals. Administration of cysteamine decreased significantly the lipid peroxidation. This suggests that cysteamine played an antioxidant role in oxidative stress induced by the EPD.

Hara and Shiota [26] reported that pancreas weight, protein and RNA contents were increased with an increased level of protein intake. We also obtained similar results. Our data indicates that ingestion of excessive protein diet led to a significant increase of pancreas/body weight, and protein, DNA and RNA contents were increased by 21.69%, 20.37% and 72.14% in pancreas of the EPD-fed mice compared with the NPD-fed mice. This result indicates that an excessive protein diet could stimulate cell differentiation and growth, and promote synthesis of protein, DNA and RNA in pancreas. A possible reason for this result is that ingestion of an excessive protein diet needs a large number of digestive enzymes synthesized and secreted by the pancreas in order to ensure the normal digestion and absorption of protein in the organism. However, the synthesis of DNA and RNA needs a large amount of ATP to synthesize purine and pyrimidine and activate amino acids, and the ATP-generating process is accompanied by free radical production, so that oxygen free radicals are excessively produced, which is the possible reason for oxidative stress induced by the excessive protein diet.

It is reported that a high protein diet is detrimental to renal functions and insulin sensitivity [27–29], and related to prostate cancer and calcium oxalate nephrolithiasis [30,31]. Therefore, ingestion of excessive protein can cause adverse effects to the healthy population. The endoplasmic reticulum system of endocrine and exocrine glands in the pancreas is subjected to free radical attack, so the pancreas is particularly sensitive to peroxidation damage [32], which is obvious from the decreased digestive enzyme activities and the increased hormones levels in the EPD group of our study.

Digestive enzymes secreted by exocrine gland directly affect digestion and absorption of the various nutrients, thus, they are often used as reliable indicators for measuring exocrine glandular function of the pancreas. Our data showed a downtrend of digestive enzyme activities in mice fed with the EPD, and significant decrease of enzyme activities with an increase of free radicals levels. Administration of cysteamine to EPD-fed mice significantly increased the activity of digestive enzymes. Therefore, we presume that through inducing excessive production of oxygen free radicals, excessive protein may attack the cell membrane and lead to membrane damage, mainly including lipid peroxidation, DNA damage and protein degradation, which cause functional damage of acinar cells and decline of secretory function and finally results in pancreatic exocrine dysfunction. In addition, the reason that digestive enzyme activity was lowered may be related to regulation of somatostatin (SS) upon excessive production of oxygen free radicals, because SS could indirectly inhibit the production of oxygen free radicals by weakening secretion function of pancreatic gland alveolus and reducing the need of ATP, in order to maintain the balance between oxidant and antioxidant.

Insulin and SS secreted by islet cell are related to some current diseases with high incidence, for example, diabetes. Insulin can reflect the reserve and secretion function of islet cell. SS is an inhibitory regulation hormone, so it could reduce oxygen free radicals produced due to energy consumption in the absorption process by inhibiting exocrine pancreatic function [33]. In the present study, levels of insulin and somatostatin were markedly higher in mice fed with the EPD compared with those fed with the NPD, which indicates that protein levels in the diet has an effect on secretion function of islet cells [34]. In addition, we also found that with an increase in oxygen free radical contents in the pancreas, hormone levels showed a trend to increase; thus, we presume that on one hand, excessive free radicals may lead to oxidative damage of islet cell, while on the other hand, increase of oxygen free radicals may induce secretion of SS, which is attributed to SS’regulatory effect.

## 5. Conclusions

In summary, these findings suggest that ingestion of excessive protein could result in oxidative damage of pancreas function through inducing oxidative stress in the pancreas.

## Figures and Tables

**Table 1.** Composition of the diets (g/kg) a.

**Ingredient**	**Group 1**	**Group 2**	**Group 3**
soybean protein b	200	600	600
Corn starch c	580	220	220
Sucrose	60	20	20
Soybean oil d	50	50	50
Cellulose powder c	50	50	50
Mineral mixture e	40	40	40
Vitamin mixture f	20	20	20
Cysteamine c			0.06

a The diets were semipurified, isoenergetic (16.20 MJ/kg).

b Shanghai, China.

c Wuxi, China.

d The commercial product (50 g/kg) provides 11.81% of energy. The soybean oil provides the following fatty acids: 14:0, traces; C16:0, 10.3; C16:1 ω-7, 0.1; C18:0, 3.9; C18:1 ω-7 + ω-9, 22.1; C18:2 ω-6, 54.8; C18:3 ω-3, 7.5; C20:0, 0.4; C20:1 ω-9 + ω-11, 0.2; C22:0, 0.4; C22:5 ω-3, traces; C24:0, traces; sum of saturated fatty acids (S), 15; sum of monounsaturated, 22.4; sum of polyunsaturated fatty acids (P), 84.7; P/S, 5.65; Σω-6/Σω-3, 7.3.

e The salt mixture provides the following amounts (g/kg diet^−1^): Ca, 4; K, 2.4; Na, 1.6; Mg, 0.4; Fe, 0.12; trace elements: Mn, 0.032; Cu, 0.005; Zn, 0.018; Co, 0.00004; I, 0.00002.

f The vitamin mixture provides the following amounts (mg/kg diet^−1^): retinol, 12; cholecalciferol, 0.125; thiamin, 40; riboflavin, 30; pantothenic acid, 140; pyridoxine, 20; inositol, 300; cyanocobalamine, 0.1; ascorbic acid, 1600; (dL) α-tocopherol, 340; menadione, 80; nicotinic acid, 200; paraaminobenzoic acid, 100; folic acid, 10; biotin, 0.6; choline, 2720. Group 1 (normal protein diet [NPD]), a normal diet containing 20% soy protein; Group 2 (excessive protein diet [EPD]), an excessive protein diet containing 60% soy protein; Group 3, excessive protein diet plus 0.06 g/kg cysteamine.

**Table 2.** Viscera indices in mice.

**Group**	**Pancreas/body weight**	**Liver/body weight**	**Kidney/body weight**	**Spleen/body weight**
Group 1	3.8 ± 0.5 ^a^	40 ± 2	12 ± 0.9	4 ± 0.4
Group 2	5.8 ± 0.3 ^b^	40 ± 5	11 ± 0.1	4 ± 0.7
Group 3	3.5 ± 0.7 ^a^	50 ± 5	14 ± 1.1	5 ± 0.9

Values are mean ± SD, *n* = 10. Within an array, values without a common superscript significantly differ, *P* < 0.05. Group 1 (normal protein diet [NPD]), a normal diet containing 20% soy protein; Group 2 (excessive protein diet [EPD]), an excessive protein diet containing 60% soy protein; Group 3, excessive protein diet plus 0.06 g/kg cysteamine.

**Table 3.** MDA content, SOD and GSH-Px activities and T-AOC in pancreas.

**Group**	**MDA (nmol/mg prot)**	**SOD (U/mg prot)**	**GSH-Px (U/mg prot)**	**T-AOC (U/mg prot)**
Group 1	3.85 ± 0.69 ^a^	101.44 ± 1.78 ^c^	100.362 ± 1.22	3.00 ± 0.04 ^c^
Group 2	10.98 ± 2.29 ^c^	70.92 ± 4.05 ^a^	91.768 ± 2.53	1.18 ± 0.07 ^a^
Group 3	5.99 ± 0.12 ^b^	85.77 ± 4.34 ^b^	97.689 ± 1.69	2.16 ± 0.06 ^b^

Values are mean ± SD, *n* = 10. Within an array, values without a common superscript significantly differ, *P* < 0.05. Group 1 (normal protein diet [NPD]), a normal diet containing 20% soy protein; Group 2 (excessive protein diet [EPD]), an excessive protein diet containing 60% soy protein; Group 3, excessive protein diet plus 0.06 g/kg cysteamine.

**Table 4.** Protein, DNA and RNA content in pancreas (mg/g).

**Group**	**Protein**	**DNA**	**RNA**
Group 1	50.66 ± 3.26 ^a^	1.08 ± 0.10 ^a^	2.01 ± 0.16 ^a^
Group 2	61.65 ± 5.13 ^b^	1.30 ± 0.12 ^b^	3.46 ± 0.33 ^b^
Group 3	51.96 ± 3.56 ^a^	1.12 ± 0.10 ^a^	2.18 ± 0.21 ^a^

Values are mean ± SD, *n* = 10. Within an array, values without a common superscript significantly differ, *P* < 0.05. Group 1 (normal protein diet [NPD]), a normal diet containing 20% soy protein; Group 2 (excessive protein diet [EPD]), an excessive protein diet containing 60% soy protein; Group 3, excessive protein diet plus 0.06 g/kg cysteamine.

**Table 5.** Amylase, lipase and trypsin activities in pancreas (U/mg prot).

**Group**	**Amylase**	**Lipase**	**Trypsin**
Group 1	0.65 ± 0.02 ^b^	57.13 ± 1.32 ^b^	154.43 ± 1.81 ^c^
Group 2	0.43 ± 0.01 ^a^	40.41 ± 0.89 ^a^	67.64 ± 0.64 ^a^
Group 3	0.58 ± 0.01 ^a^	52.79 ± 2.71 ^b^	84.95 ± 2.97 ^b^

Values are mean ± SD, *n* = 10. Within an array, values without a common superscript significantly differ, *P* < 0.05. Group 1 (normal protein diet [NPD]), a normal diet containing 20% soy protein; Group 2 (excessive protein diet [EPD]), an excessive protein diet containing 60% soy protein; Group 3, excessive protein diet plus 0.06 g/kg cysteamine.

**Table 6.** Somatostatin and insulin levels in pancreas.

**Group**	**Somatostatin (U/mg prot)**	**Insulin (pg/mg prot)**
Group 1	47.82 ± 1.33 ^a^	46.70 ± 1.76 ^a^
Group 2	83.11 ± 2.20a ^c^	55.91 ± 2.93 ^c^
Group 3	63.49 ± 2.08 ^b^	50.85 ± 2.43 ^b^

Values are mean ± SD, n = 10. Within an array, values without a common superscript significantly differ, P < 0.05. Group 1 (normal protein diet [NPD]), a normal diet containing 20% soy protein; Group 2 (excessive protein diet [EPD]), an excessive protein diet containing 60% soy protein; Group 3, excessive protein diet plus 0.06 g/kg cysteamine.
